# Supplementary figures and images for: Taxonomic characterization and cytotoxic potential of Vietnamese Ganoderma ellipsoideum against human breast cancer MCF-7 cells
Source: PLoS One. 2025 Nov 14;20(11):e0336024. doi: 10.1371/journal.pone.0336024 (PMC12617861; doi:10.1371/journal.pone.0336024)

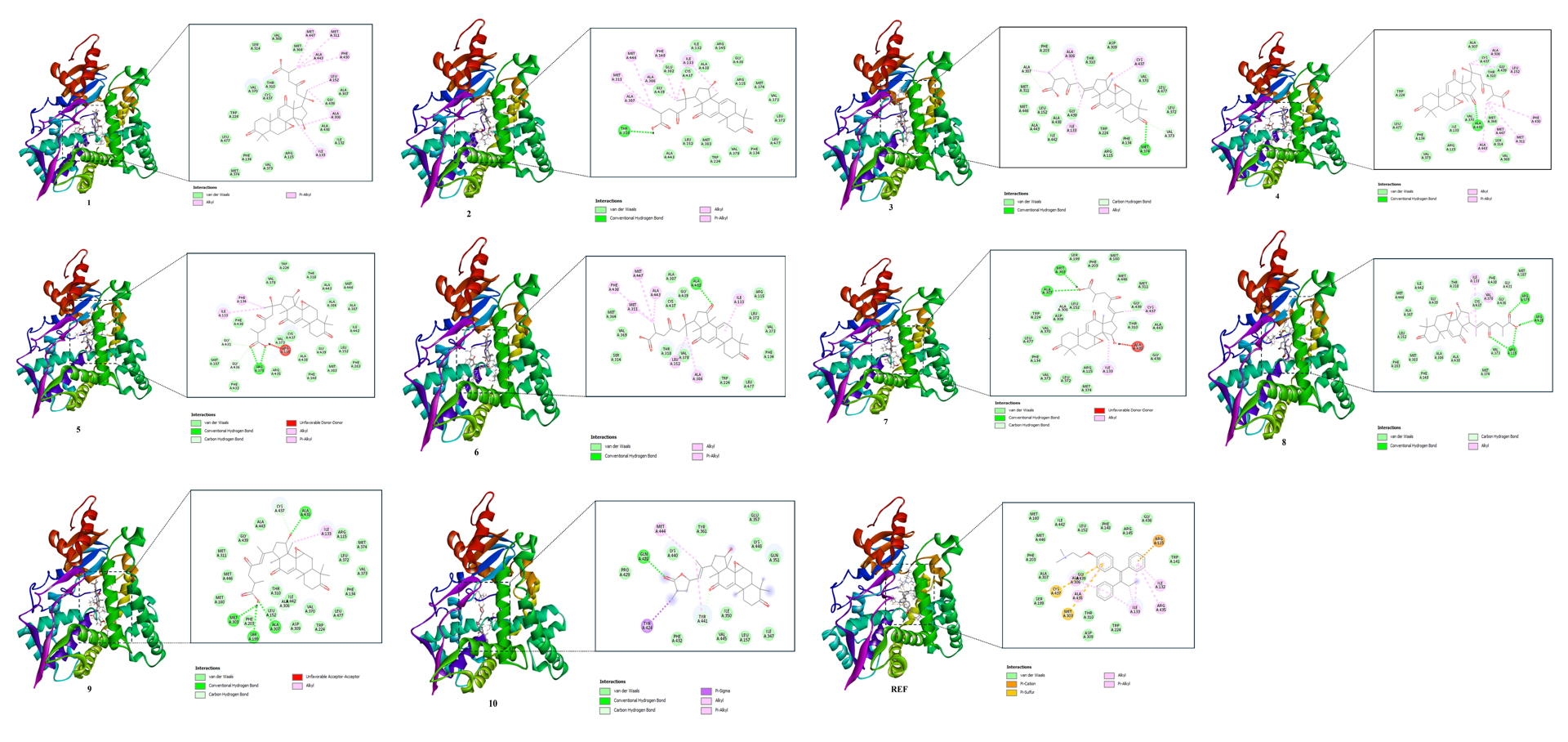

Supplement: S1 Fig — (PNG) [file pone.0336024.s001.png]

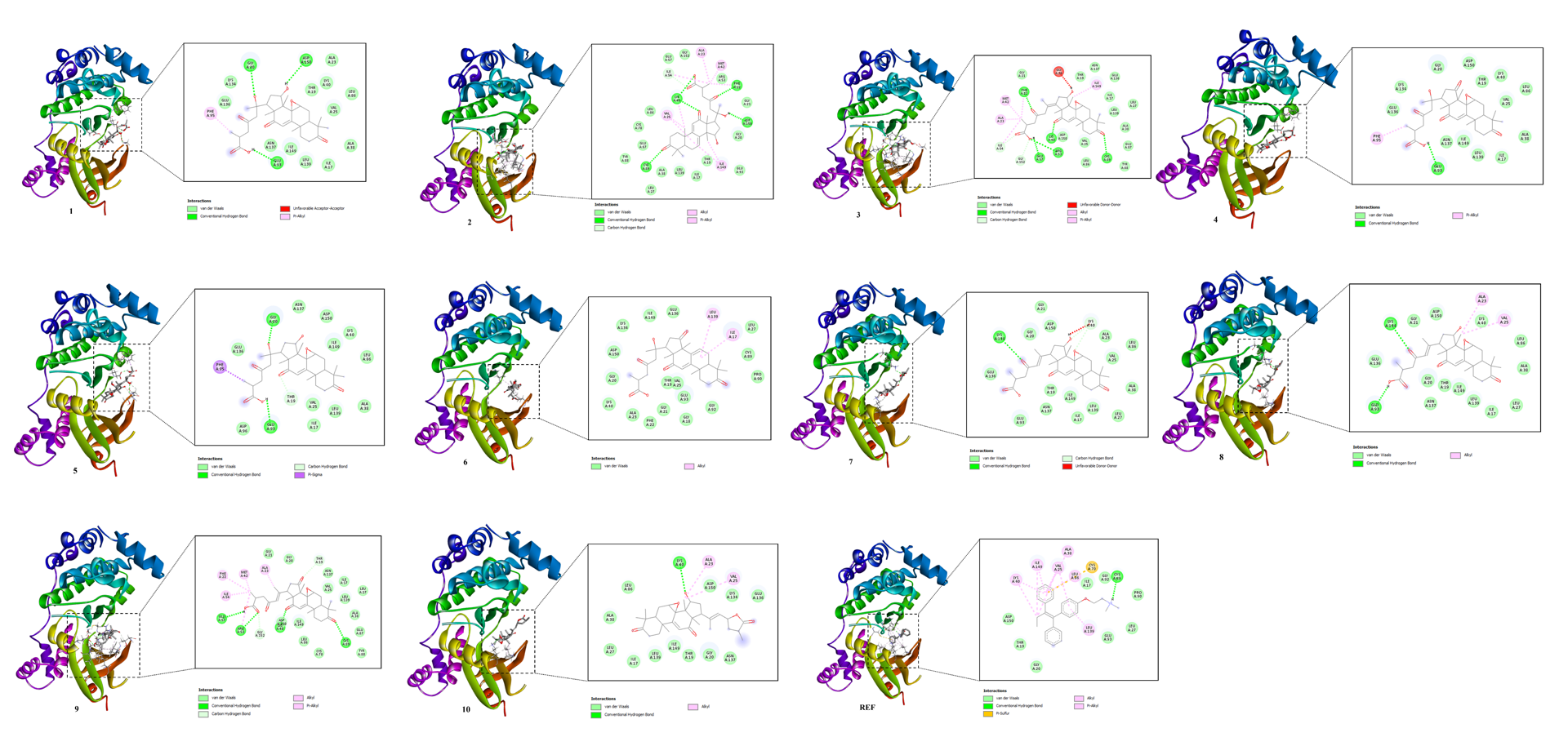

Supplement: S2 Fig — (PNG) [file pone.0336024.s002.png]

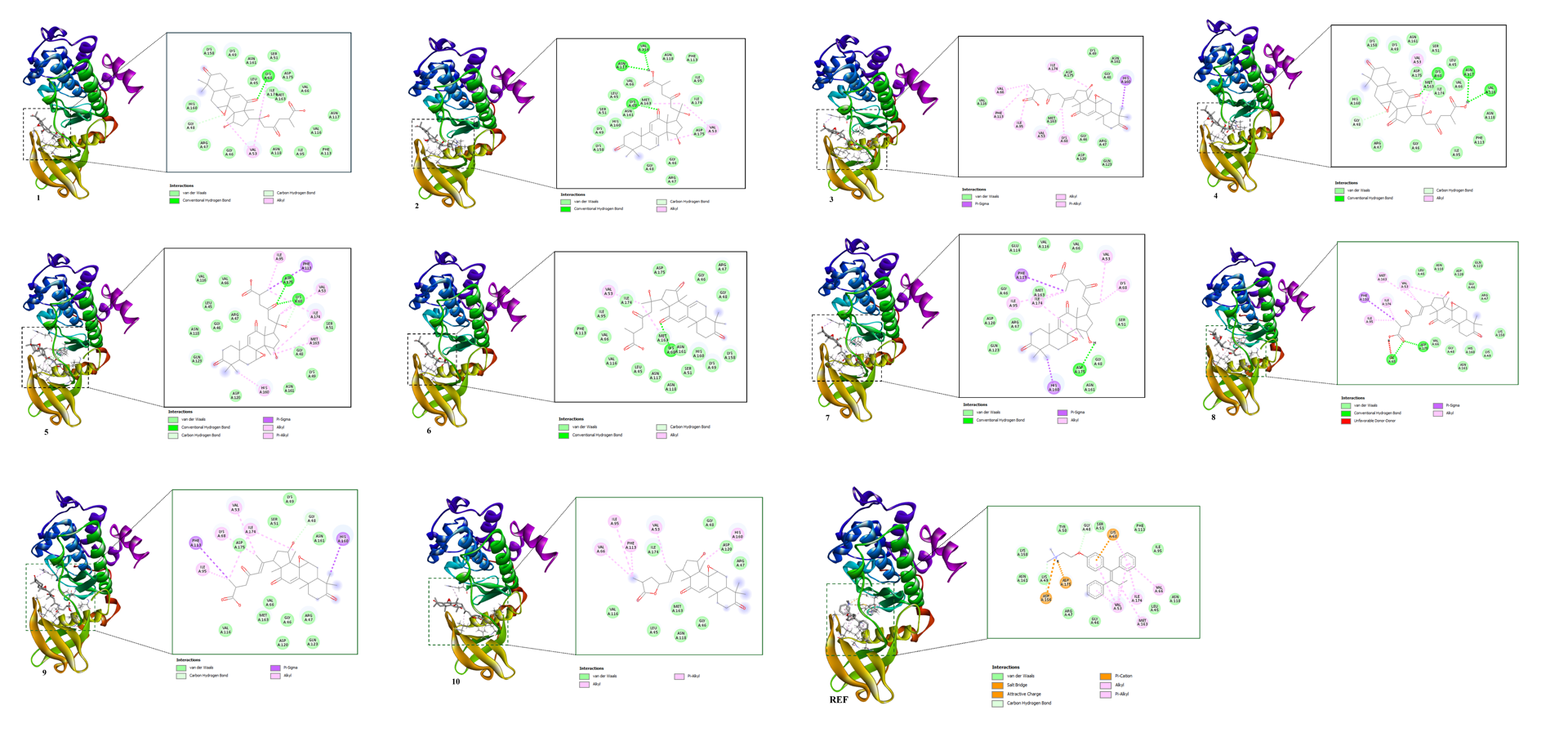

Supplement: S3 Fig — (PNG) [file pone.0336024.s003.png]

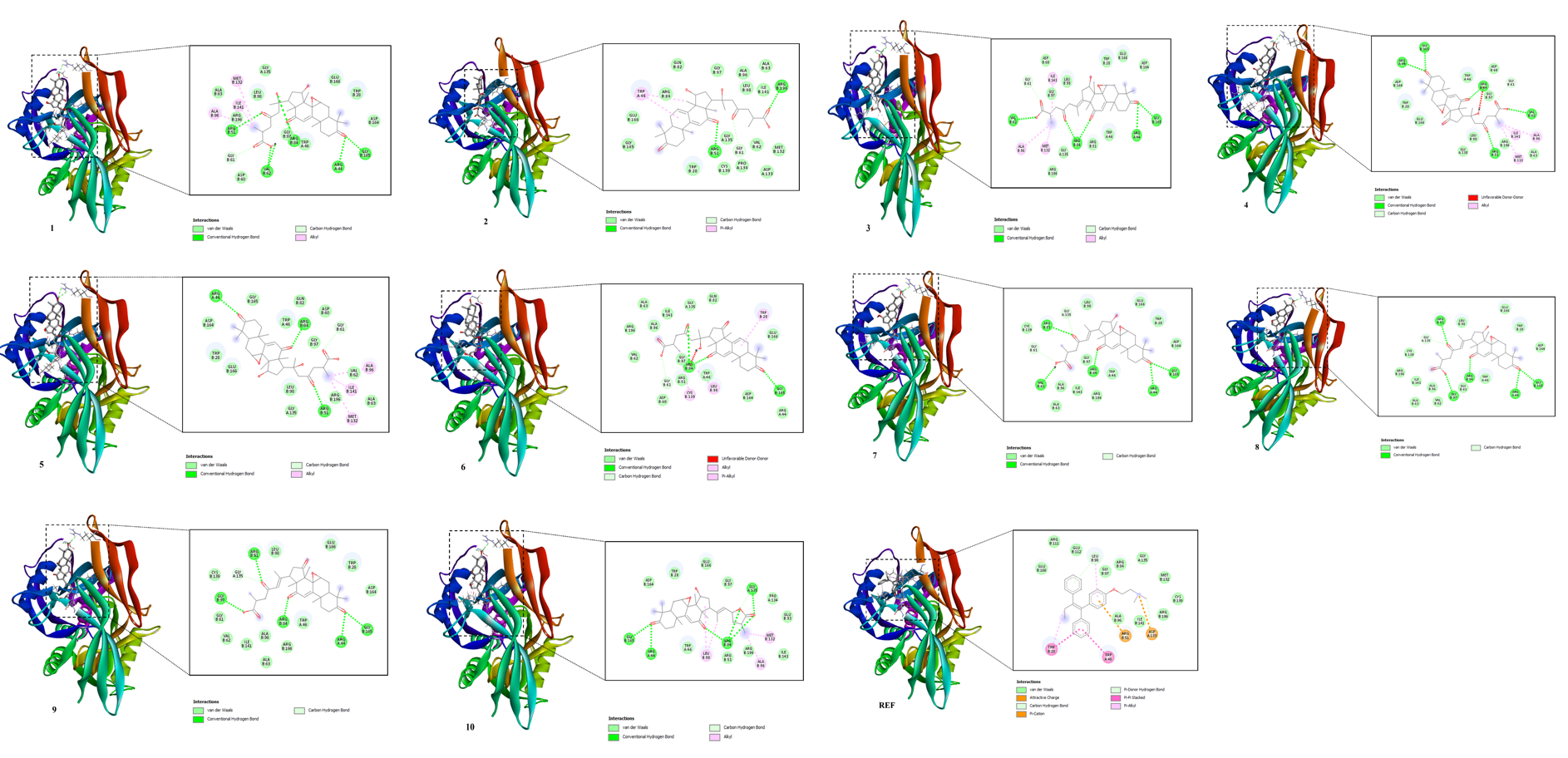

Supplement: S4 Fig — (PNG) [file pone.0336024.s004.png]
