# Supplementary material for: Taxonomic characterization and cytotoxic potential of Vietnamese Ganoderma ellipsoideum against human breast cancer MCF-7 cells
Source: PLoS One. 2025 Nov 14;20(11):e0336024. doi: 10.1371/journal.pone.0336024 (PMC12617861; doi:10.1371/journal.pone.0336024)
Supplement: Attachment — Submitted filename: PONE-D-25-37431 Response to Reviewers.docx [file pone.0336024.s006.docx]

**Response Journal Requirements:**

1. Please ensure that your manuscript meets PLOS ONE's style requirements, including those for file naming. The PLOS ONE style templates can be found at

<https://journals.plos.org/plosone/s/file?id=wjVg/PLOSOne_formatting_sample_main_body.pdf>

[https://journals.plos.org/plosone/s/file?id=ba62/PLOSOne_formatting_sample_title_authors_affiliations.pdf](https://track.editorialmanager.com/CL0/https:%2F%2Fjournals.plos.org%2Fplosone%2Fs%2Ffile%3Fid=ba62%2FPLOSOne_formatting_sample_title_authors_affiliations.pdf/1/010f01988d472ebb-ca989892-927b-4d82-af31-e04931f77322-000000/fvSWTcFYVY80yVAUbwPSOy_5WmoaBDFFeMR3tOQEDoE=223).

*Answer:* We editted that the manuscript met PLOS ONE's style requirements.

2. We noticed you have some minor occurrence of overlapping text with the following previous publication(s), which needs to be addressed:

<https://www.researchsquare.com/article/rs-2421198/v1>

<https://www.tjnpr.org/index.php/home/article/view/5237>

*Answer:*  We have cited two publications, listed as references 34 and 60.

3. Thank you for stating the following financial disclosure: “Scholarship Program of the Vingroup Innovation Foundation (VINIF), Vingroup Big Data Institute (VINBIGDATA) (grant number: code VINIF.2020.TS.68).” Please state what role the funders took in the study.  If the funders had no role, please state: "The funders had no role in study design, data collection and analysis, decision to publish, or preparation of the manuscript." If this statement is not correct you must amend it as needed. Please include this amended Role of Funder statement in your cover letter; we will change the online submission form on your behalf.

*Answer:* Thanks, we revised the problem, please see our funding statement.

4. Please provide a complete Data Availability Statement in the submission form, ensuring you include all necessary access information or a reason for why you are unable to make your data freely accessible. If your research concerns only data provided within your submission, please write "All data are in the manuscript and/or supporting information files" as your Data Availability Statement.

*Answer:*

5. Thank you for stating the following in the Acknowledgments Section of your manuscript:

“Trang Thi Thu Nguyen was funded by the Vingroup Joint Stock Company and supported by the PhD Scholarship Program of the Vingroup Innovation Foundation (VINIF), Vingroup Big Data Institute (VINBIGDATA) (grant number: code VINIF.2020.TS.68).”

We note that you have provided additional information within the Acknowledgements Section that is not currently declared in your Funding Statement. Please note that funding information should not appear in the Acknowledgments section or other areas of your manuscript. We will only publish funding information present in the Funding Statement section of the online submission form.

Please remove any funding-related text from the manuscript and let us know how you would like to update your Funding Statement. Currently, your Funding Statement reads as follows:

“Scholarship Program of the Vingroup Innovation Foundation (VINIF), Vingroup Big Data Institute (VINBIGDATA) (grant number: code VINIF.2020.TS.68).”

Please include your amended statements within your cover letter; we will change the online submission form on your behalf.

*Answer:* We revised funding-related text from the manuscript.

**Response to Additional Editor Comments**

1. It has been noted that the manuscript's primary focus is on the identification and characterization of Ganoderma ellipsoideum using in silico methods, with very little experimental work on cytotoxicity. Please revise the title to better reflect the core hypothesis and content of your research.

*Answer:* Thank you very much for this insightful comment. We fully understand and appreciate your suggestion to align the title more closely with the core hypothesis and content of the study, particularly regarding the cytotoxic potential against MCF-7 breast cancer cells. However, in this manuscript, our primary objective was to identify, classify, and report the presence of *Ganoderma ellipsoideum* in Vietnam for the first time. Although this species has been described in other regions, its discovery in Vietnam represents a noteworthy contribution to the national mycological and pharmacological biodiversity.

The in vitro cytotoxicity assay presented here serves as a preliminary step to explore the anticancer potential of this fungal species. We acknowledge that further in-depth studies are needed to fully elucidate its bioactivity. These investigations are currently in progress and will be the focus of future publications. We hope that the current title, or a slightly adjusted one, can still reflect the scientific contribution and scope of this work, which emphasizes both the taxonomic characterization and the initial screening for cytotoxic activity.

2. Please include a detailed description of the sample preparation and extraction methods in the section of Materials and Methods.

*Answer:* We added a detailed description of the sample preparation and extraction methods in the section of Materials and Methods.

3. To validate you’re in silico results, please perform a Western blotting experiment for protein identification

*Answer:* Thank you for the valuable suggestion. We fully acknowledge that validating in silico results requires complementary experimental evidence, including Western blotting and even in vivo studies. However, at this stage, we are currently facing limitations in terms of laboratory equipment and research funding, which prevent us from performing these essential experiments. We sincerely hope to overcome these constraints in the near future and conduct further studies to clarify and confirm the potential cytotoxic effects of the compound against human breast cancer MCF-7 cells.

4. Cytotoxicity experiment results in the Tables 3 and 4 into a single table for clarity.

*Answer:* We merged Cytotoxicity experiment results in the Tables 3 and 4 into a single table with name "Table 3".

5. The resolution of Figures 3, 4, 5, and 6 is too low. Please submit high-resolution versions of these images.

*Answer:* We sincerely apologize for the low resolution of Figures 3, 4, 5, and 6. These images were captured directly from the original docking software outputs, and unfortunately, higher-resolution versions are currently unavailable due to software limitations. Therefore, we kindly request to retain the current versions of these figures in the revised manuscript.

6. The manuscript requires an extensive revision for grammar, syntax, and overall clarity. Please consider having it proofread by a native English speaker or a professional editing service.

*Answer:* Thank you very much for your suggestion regarding grammar and clarity. We have thoroughly revised the manuscript to correct typographical, grammatical, and syntactical issues. The updated version has been carefully proofread to improve the overall readability and language quality. Please review the revised main manuscript file for confirmation of these improvements.

**Response to Reviewers**

| REVIEWER 1 COMMENT | AUTHOR RESPONSE | PAGE NUMER |
| --- | --- | --- |
| There's a small typo in the phrase “Maximum Likehood” — it should be corrected to “Maximum Likelihood.” | We corrected from “Maximum Likehood”to “Maximum Likelihood.” | 10 |
| In the Results section, it would be helpful to write out “Maximum Likelihood (ML)” the first time it's mentioned, even though it's already explained in the Methods. | We wrote out “Maximum Likelihood (ML)” the first time it’s mentioned in the Results section. | 10 |
| The phrase “anti cancer” should be written as one word: “anticancer.” | We wrote the phrase “anti cancer” to one word “anticancer”. | 2 |
| the word “basidicarps” appears to be misspelled — the correct term is “basidiocarps.” | We corrected the term “basidicarps” to “basidiocarps”. | 11 |
| Missing articles (a / the): There are a couple of places where the article “a” is missing. For example: • “...a previous study of Jiao and coworkers (2013)” • “...as a potential inhibitor of anti-breast cancer targets” | We added "a" into   - “... previous study of Jiao and coworkers (2013)”. - “...as potential inhibitor of anti-breast cancer targets” | 12  18 |
| REVIEWER 2 COMMENT | AUTHORS RESPONSE | PAGE NUMER |
| This study has several notable strengths. It reports Ganoderma ellipsoideum in Vietnam for the first time, confirmed through both morphological characterization and molecular phylogenetic analysis, thereby expanding the known distribution and taxonomy of the species. The work integrates taxonomy, in vitro cytotoxicity testing, and in silico molecular docking, linking biodiversity research to pharmacological potential. The focus on well-established breast cancer–related targets (HPA, MELK, CK2α, NUDT5) enhances biomedical relevance, and the identification of triterpenoids with predicted binding affinities exceeding that of tamoxifen highlights promising anticancer potential. The study also provides transparent sequence data deposition and applies statistical rigor, laying a strong foundation for future compound isolation, mechanistic work, and in vivo validation.  At the same time, several limitations should be acknowledged. Cytotoxicity was assessed only in a single breast cancer cell line (MCF-7) without comparison to normal cells, preventing evaluation of cancer selectivity. The findings are based solely on in vitro assays and in silico predictions, with no in vivo validation or direct biochemical confirmation of the predicted targets. The active compounds were not isolated or quantified from the tested fractions, and their individual contributions to the observed cytotoxicity remain unverified. Chemical composition of extracts was not characterized, and results may vary with different extraction batches or environmental conditions of fungal growth. Docking and ADMET analyses were predictive only and may not reflect biological behavior in living systems. Finally, the study is based on specimens collected from a single location, which may limit generalizability to other populations.  Given these limitations, the conclusions should be presented more modestly, or supported by additional experiments in future work. Specifically, including multiple cancer cell lines, incorporating non-transformed control cells to assess selectivity, and performing direct biochemical confirmation of predicted molecular targets, such as via western blotting, would strengthen the link between molecular docking predictions and observed biological activity. | Thank you for your comments. We have added a paragraph on the study’s limitations to the Conclusion section.  "Although *G. ellipsoideum* was first reported in China in 2018 and has since been cultivated in Thailand, our study is the first to document its discovery and distribution in Vietnam, specifically within the Ea So Nature Reserve of Dak Lak Province. Due to the limited number of natural fruiting bodies collected, compound isolation from Vietnamese samples was not yet possible. Instead, we prepared 70% ethanol extracts and fractionated extracts (*n*-hexane, ethyl acetate, and water) to evaluate their cytotoxicity against MCF-7 human breast cancer cells. To our knowledge, this is also the first study worldwide to assess the *in vitro* cytotoxic effects of this mushroom species on human breast cancer cells. Notably, we further investigated the inhibitory potential of its triterpenoids using *in silico* models targeting four key proteins associated with breast cancer (HPA, MELK, CK2α, and NUDT5). These findings provide a valuable foundation for future research, including evaluations across additional breast cancer cell lines, compound isolation, *in vivo* experiments, and gene expression studies (e.g., Western blot) to comprehensively assess the anti-breast cancer potential of *G. ellipsoideum*." | 22 |
